# Supplementary material for: Dissection of the Fgf8 regulatory landscape by in vivo CRISPR-editing reveals extensive intra- and inter-enhancer redundancy
Source: Nat Commun. 2021 Jan 19;12:439. doi: 10.1038/s41467-020-20714-y (PMC7815712; doi:10.1038/s41467-020-20714-y)
Supplement: Supplementary file 1 — Supplementary Information [file 41467_2020_20714_MOESM1_ESM.pdf]

Supplementary information

Dissection of the *Fgf8* regulatory landscape by *in vivo* CRISPR-editing reveals extensive intra- and inter-enhancer redundancy

Hörnblad, A.[1, 2, 3, 4], Bastide, S.[1, 2, 3,*,#], Langenfeld, K.[1,*], Langa, F.[5], Spitz, F. [1, 2, 3,#]

[1] Developmental Biology Unit, EMBL, Meyerhofstrasse 1, Heidelberg 69117, Germany.

[2] (Epi)genomics of animal Development Unit, Department of Developmental and Stem Cell Biology, Institut Pasteur, 75015 Paris, France

[3] CNRS, UMR3738.

[4] Umeå Centre for Molecular Medicine, Umeå University, 90187, Umeå, Sweden.

[5] Mouse Genetics Engineering, Center for Innovation & Technological Research, Institut Pasteur, 75015 Paris, France

* These authors contributed equally

[#] Present address: Department of Human Genetics, The University of Chicago, Chicago IL, USA

Supplementary Figures 1-8

Supplementary Tables 1-6

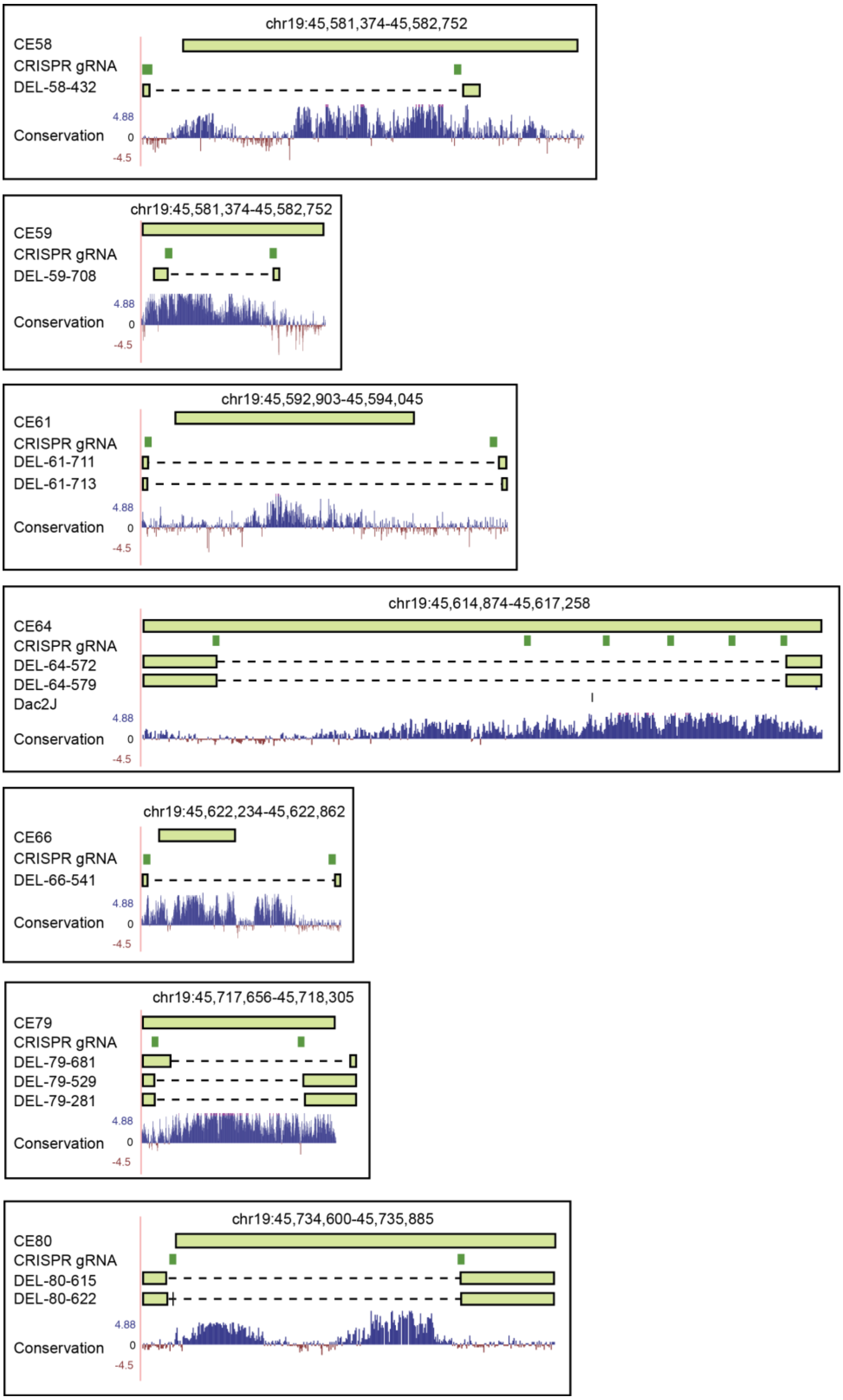

Supplementary Figure 1. Genomic coordinates of the seven putative enhancers. Upper row depicts mouse sequences homologous to human sequences with reported enhancer activity. Green boxes indicate CRISPR gRNAs used to create enhancer deletions. Dashed lines depict the location of the deletions for all lines used. DEL79-281 indicates the deletion of the DEL79-80 line that was generated over DEL80-622.

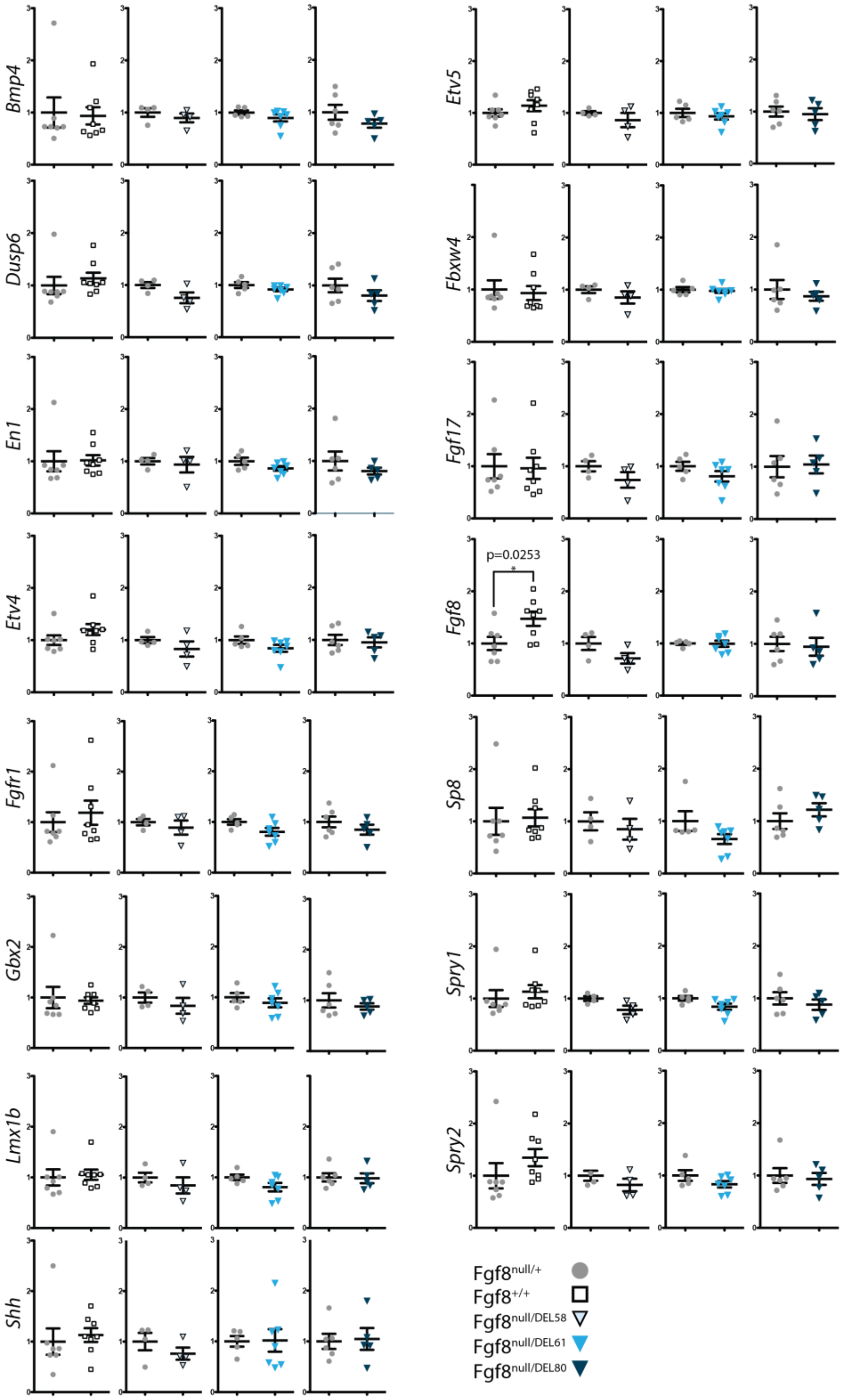

Supplementary Figure 2. Gene expression levels are maintained in AER enhancer mutants. RT-qPCR analysis of e10.5 forelimb in *Fgf8*$^{null/+}$, WT (n=7), DEL58 (n=4), DEL61 (n=5) and DEL80 (n=5) embryos. Relative expression of indicated mRNA as compared to heterozygous *Fgf8*$^{null/+}$ littermate controls. Expression was normalized to average of Hprt1 and PGK1 mRNA. Individual data points as well as mean ± SEM are indicated. *p<0.05 (two-tailed Student's *t*-test).

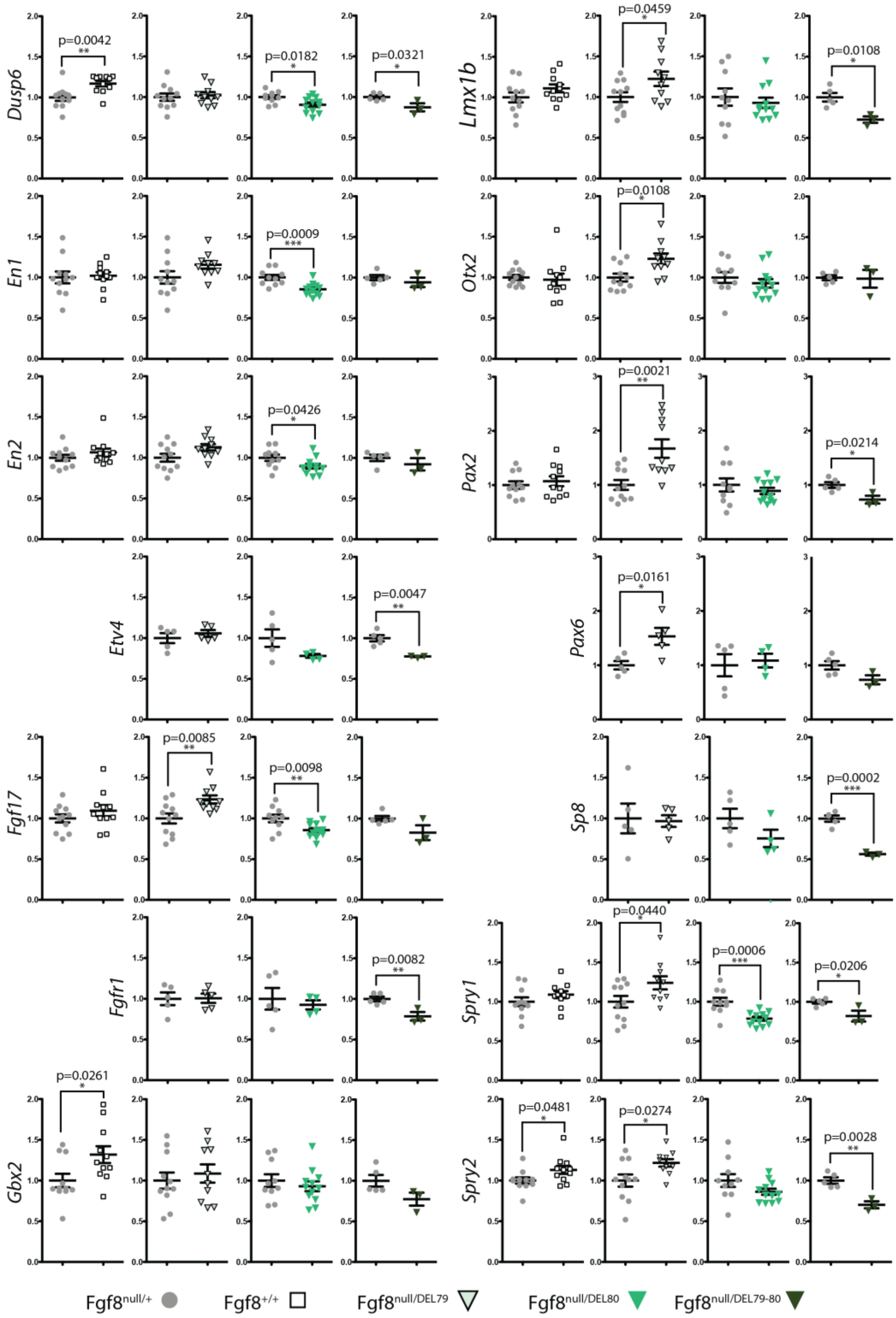

Supplementary Figure 3. Minor changes in gene expression levels accompany DEL79. DEL80 and DEL79-80 mutants. RT-qPCR analysis of e10.5 dissected MHB region in *Fgf8*$^{null/+}$ (n=11, 5/11, 5/10, 5), WT (n=11) , DEL79 (n=5/10), DEL80 (n=4/12) and DEL79-80 (n=3) embryos. Relative expression of indicated mRNA as compared to heterozygous *Fgf8*$^{null/+}$ littermate controls. Expression was normalized to average of Hprt1 and PGK1 mRNA. Individual data points as well as mean ± SEM are indicated. *p<0.05, **p<0.01, ***p<0.001 (two-tailed Student's *t*-test).

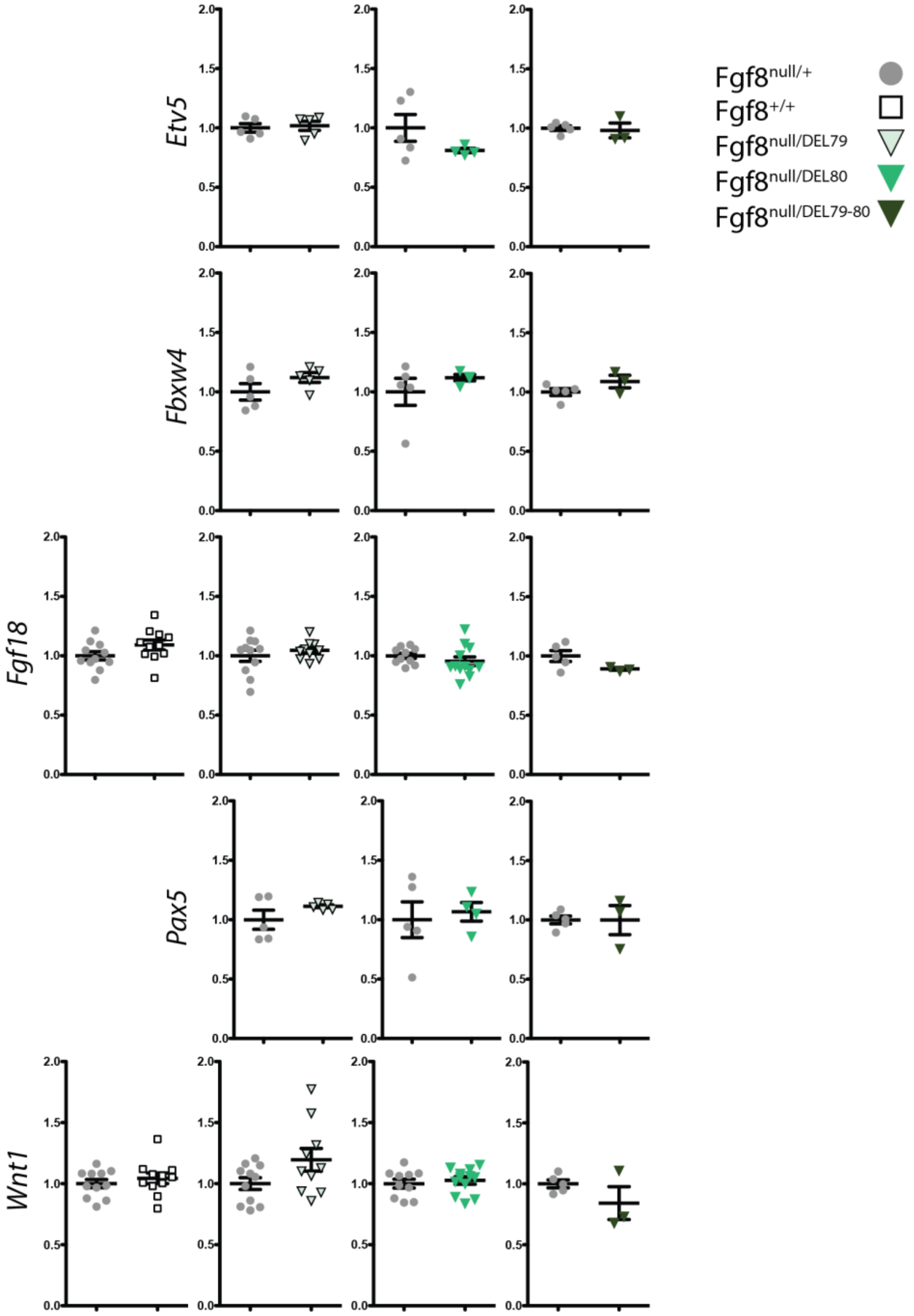

Supplementary Figure 4. Genes with no significant expression changes in any of DEL79. DEL80 and DEL79-80 mutants. RT-qPCR analysis of e10.5 dissected MHB region in *Fgf8*$^{null/+}$ (n=11, 5/11, 5/10, 5), WT (n=11), DEL79 (n=5/10), DEL80 (n=4/12) and DEL79-80 (n=3) embryos. Relative expression of indicated mRNA as compared to heterozygous *Fgf8*$^{null/+}$ littermate controls. Expression was normalized to average of Hprt1 and PGK1 mRNA. Individual data points as well as mean ± SEM are indicated.

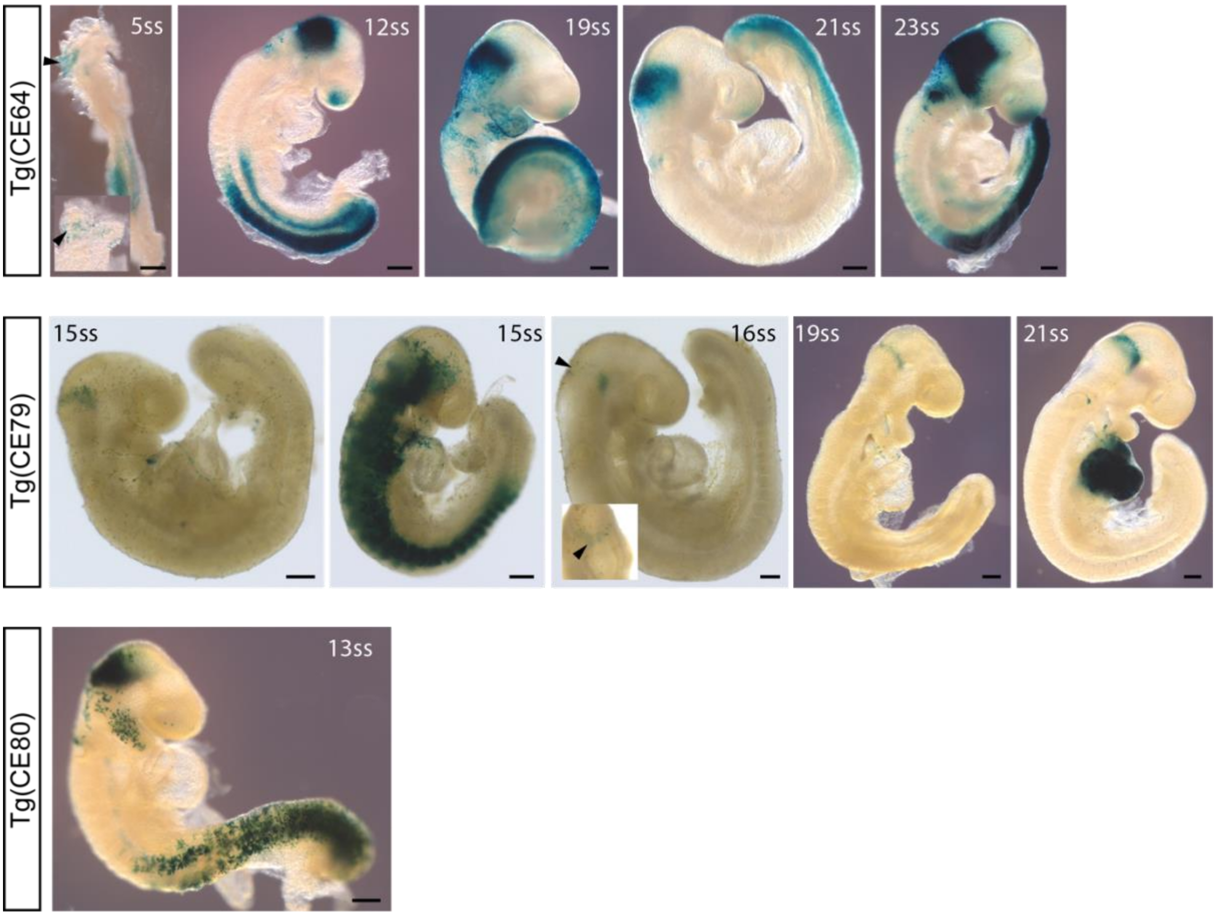

Supplementary Figure 5. Transgenic enhancer activity of MHB enhancers. Only CE64 drives reporter expression at earliest stages of MHB specification (2 out of 2 embryos, e8-8.5, 5 and 12 somites respectively). At e9.0-e9.5 (ss13-23) all enhancers display some activity in the MHB (CE64: 3 out of 6, CE79: 5 out of 6, CE80: 1 out of 3). Embryos lacking activity are not shown. Arrowhead indicate MHB region displayed in dorsal view in inset picture of Tg(CE64) and Tg(CE79) embryo. Scalebar is 200µm.

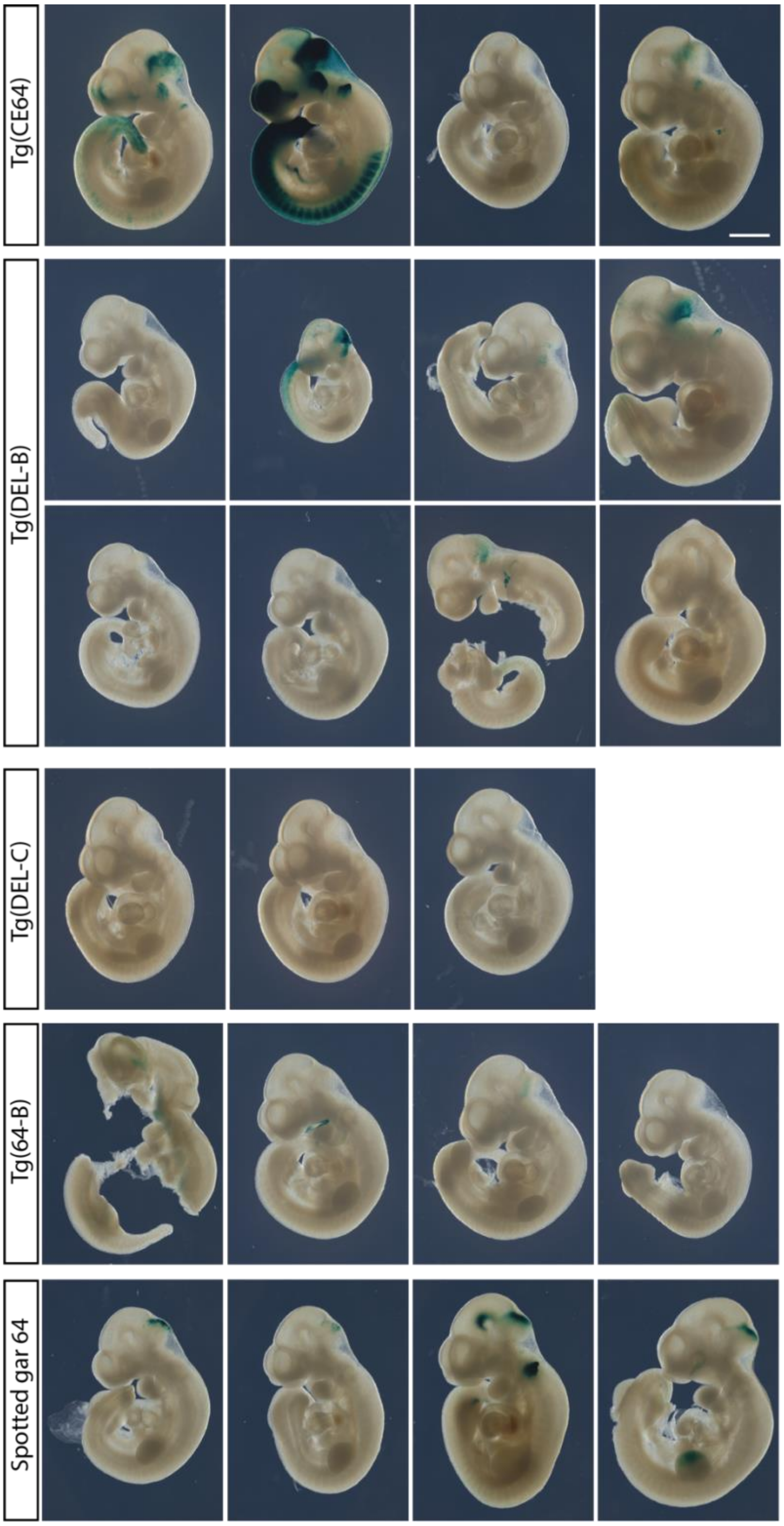

Supplementary Figure 6. Transgenic enhancer activity of mouse CE64 and spotted gar CE64. Compilation of all transgenic embryos harvested for Tg(CE64), Tg(DEL-B), Tg(DEL-C), Tg(64-B) and Tg(Spotted gar64B) . Expression is absent in the MHB of Tg(DEL-B), Tg(DEL-C) and Tg(64-B) embryos while all Tg(Spotted gar 64B) embryos display expression in the MHB region. Note also the hindbrain expression present in 4 of the Tg(DEL-B) embryos. Scalebar is 1mm.

Block#1

Human	GGACAACAAGGAAAATCGGCTG-GCCCATTGT
Mouse	GGACAACAAGGAAAATCGGCTG-GTCCATTGT
Pika	GGACAACAAGGAAAATCGGCTG-GCCTATTGT
Pig	GGACAACAAGGAAAATCGGCTG-GCCCATTGT
Dog	GGACAACAAGGAAAATCGGCGG-GCCCATTGT
Megabat	GGACAACAAGAAAAATCGGCTG-GCCCATTGT
Shrew	GGACAACAAGGAAAATCGGCCA-GCCCATTGT
Elephant	GGACAACAAGGAAAATCGGCTG-GCCCATTGT
Opossum	GGACAACAAGGAAAATAGGCTG-GCCCATTGT
Chicken	GAACAACAAGGAAAATATGCTG-GCCCATTGT
Lizard	GAACAACAAGGAAAATATGCTGAGTCCATTGT
Xenopus	AAACAACAAGAAAAATCTGCT--TTCTATTGT
Coelacanth	AAGCAACAAGAAAAACCTGCT--ACCCATTGT
Zebrafish	-----------------------GCTCATTGC
Spotted_gar	--------------------------------

Block#2

Human	GTGCTAAGTGAGATGAAAGGGG
Mouse	GTGCTAAGTGAGATGAAAGGGG
Pika	GTGCTAAGTGAGATGAAAGGGG
Pig	GTGCTAAGTGAGATGAAAGGGG
Dog	GTGCTAAGTGAGATGAAAGGGA
Megabat	GGTGCTAAGTGAGATGAAAGGG
Shrew	GTGCTAAGTGAGATGAAAGGGG
Elephant	GTGCTAAGTGAGATGAAAGGGG
Opossum	GTGCTAAGTGAGATGAAAGCAG
Chicken	GCACCAAGGGAGATGAAAGGGA
Lizard	ACACTATAGGAGATGAAAGGGA
Xenopus	GTACTAAGTGAGATGAAAGGGA
Coelacanth	GAGCAGGATTAAAAAAAAAGGG
Zebrafish	--GCT-----------------
Spotted_gar	----------------------

Block#3

Human	CCCGCCTGCCAATCA
Mouse	CCCGCCTGCCAATCA
Pika	CCCGCCTGCCAATCA
Pig	CCCGCCTGCCAATCA
Dog	CCCGCCTGCCAATCA
Megabat	CCCGCCTGCCAATCA
Shrew	CCCGCCTGCCAATCA
Elephant	CCCGCCTGCCAATCA
Opossum	CCCGCCTGCCAATCA
Chicken	ACCACCTGCCAATCA
Lizard	GCTGCCTGCCAATCA
Xenopus	ACAGCCTGCCAATCA
Coelacanth	CCATCCTGTCAATCA
Zebrafish	---------------
Spotted_gar	-CCACCTCCCAACCA

Block#4

```
Human              AGTTATT-AAGGTCACAATGTTAAATTCTTTTGACCTGCACCTAATCAAAGAT
Mouse              AGTTATT-AAGGTCACAATGTTAAATTCTTTTGACCTGCACCTAATCAAAGAT
Pika               AGTTATT-AAGGTCACAATGTTAAATTCTTTTGACCTGCACCTAATCAAAGAT
Pig                AGTTATT-AAGGTCACAATGTTAAATTCTTTTGACCTGCACCTAATCAAAGAT
Dog                AGTTATT-AAGGTCACAATGTTAAATTCTTTTGACCTGCACCTAATCAAAGAT
Megabat            AGTTATT-AAGGTCACAATGTTAAATTCTTTTGACCTGCACCTAATCAAAGAT
Shrew              AGTTATT-AAGGTCACCATGTTAAATTCTTTTGACCTGCACCTAATCAAAGAT
Elephant           AGTTATT-AAGGTCACAATGTTAAATTCTTTTGACCTGCACCTAATCAAAGAT
Opossum            AGTTATT-AAGGTCACAATGTTAAATTCTTTTGACCTGCACCTAATCAAAGAT
Chicken            AGTTATT-AAGGTCACGATGTGAAATTCTTTTGACCTACACCTAATCAAAGAT
Lizard             ACTTATT-AAGGTCACAATGT-AGACAATATTAACCTGAATCCAATCAAAGAT
Xenopus            AGTAAGCAAAGGTCACAATGTTACATTCTTTTGACCTGCACTTA---------
Coelacanth         AGGTATC-AA-------------------------------------------
Zebrafish          -----------------------------------------------------
Spotted_gar        -----------------------------------------------------
```

Supplementary Figure 7. MUSCLE alignments of conserved blocks in 64-C. Disruption of both block #2 and #3 together through CRISPR/Cas9 editing cause absence of MHB-derived structures while deletion of any single block does not cause phenotype.

>Spotted_gar
CAGTATAATATTCATTCCATGTATTCTCACTAGGTGTTTGTGCTTTGGTTATGCATCTACTTGTTTTCCTTCAAG
ATCTAGAGAATATAATAGAACAATGTGAACCTGTCTATACAAACAATATATGAAAATATTTGTTGGGCTTTAATG
CAGTTTGTACAGTTTATTCAATTATATTTTTCTTGTGTGGAAGAAATTTCATTTGTCATATCAAGGTCATGCTGT
ACTTTGTGCTTAAAACATTTCTAAAAATTTAATTTTAATATCCTGTCAGAACACAAAAAAGACTTAGTGATTGCT
CAGTGCACATTCTTCTACACTTGGAGTGACACAAATTAAAATAAGTTTTATTTCATAATTACTGCAGTGTCAGAT
TGAGAAGTCTTAGGCTGCATCTCAATCCTGCCTAAAGTAGGATTAGTGCAAATTCTTCCCTGGACATTTACATAT
GGGCTGTTTGCTAAATTAATTTGCTACAATGTTGACACATCCTGTCTGTACTGAGGATTTTGTAGATGTAGTGAT
CTCCCATTTATGCTAAAACATATAATCATTTTTTTAAGCGACATCCGGCAAGCCCGAGGGGTCTTTTTTTCGGTC
GTCTTTTAAAAATTCCTTCTAGATCATTTTTTCTACTTTTGACATAATGTTCAATGTGTTTATTCAGAGAAGACA
ACAGAATATTGCATGATACCTTATAGAATATTGAACAAATGTGACCATTCGTTATGGTATGTTGATAATTGTTCA
AAATACTCATGACATTTTGATTTGTCTTTCCCCAGTTGCACCCTGTGCAACATCCAGACGGTCCATAAGCCTCCA
AGAAGGCTGTGGGTTTACAACTGGTCTGAAGTCACCTGAATGCCTGTGGCCCCGAGTTGGATGGA**TGTGGTTGG**
GAGGTGGTGCTGGGACTCTGCAATAAAGGACCCTAACTAAGTGTCAAAGGGCCTGGCGCACAATGCTGAGCAGTG
GGTGGAGGTCTCCACTGACCACTCCAAAGGGAGGGGGTTCTGAGGCACTCAAGGGAGAAGCCACCCCGAGGAGAG
GCAAAAAAAAAAAGAGGAGGGGGGTGTTTTGTCCAAAACCCGTCAAGCACATCTACAAAGTTGTTGCAGGGCAAT
GCCTCTGAATTTTTCAAAAGAAAATGATTGTTTGATAAACCGCCCAGAATCACCACTTTCTCACAACGAAGGGAG
CATCACCTCTGACCTTTGTATAAGCGTACAGCGAGACTCGCTGCTGCTACCCCAAGCCCCCCCCCCACCACCCC
TCGCCTCCATTGCTCCGCCCCACTCAACCGCAAAACTTTGATGCGCGCGTCTCTCTCTCTCTCGTTTTTTTGGTC
CCTCTCGAGCTTTGTAGAGCGGGCTGCGCCTTTGGAGTCAAGAATGCAATTAGTTTTTTTTTTTTAAAAAAAAGG
CGAGGAAAAAGAGAAGCCGCAACAATAAAGCGATAACGGATAGAATGTACGTTCCCACACATTAAATATGGGGCG
TATTTTTGTTCCCTGGGAGCAGAGGGAGAGAGCAGCTTCAGACAGCAGGTGGTGTCCCAAATAACCGCCGTCAGT
ATTCCCCATCACTGCAGACTTCGTTCAAAATCCAGTTCATTAGAGACATGACCCATGCAGGTGTAACTGGCTAGA
CATTTCAGACAGAACTTTTAATAGCGCGCTTGAAAACTCATATCGGTAATGAGATGGAGTAGGCTACCCTGTGCT
AGGGGTTTATTTCTGTCTGAAAGACTGATGGTTTCACCATGCTGCCTTCACACTTCAGATCTCAAGTTTATGCCG
ATCCCAAAGAGAGTGCATAGTGTTTGTTTTTCAGCGCAAAAGTGGAGGTTTCAGGGTTGACAATTTGCTCAATGT
GTTGTTGTTCGGGTGATAAACACTGCAAAATCTTTAAAGAAAACAACGGAATTTTAAAAACTGGTGGCATTGTGA
GTGATGATGAGAATGTTCTGTGCTGATTACCGGGTTTTATTAAATTCTCCTCTGTCTGTATGCACGTGAAGGAGA
ATAAAATAATAATGAAGTTTTTCAGGCTTGTTTCGGTAGTGGCTGCTCAGATTTGTTTGTTTGGTCTTCTCTATT
ACTGGGAAAGACAAACACTTGCACTTTGTAAATGGAGTCCTTCTTAATTCTAAGCAGAAGAAATACAACATCCTT
CTTGTTGTAAGAGCTATTTGCTCTTTAGCAACCTGGAGGTAACAGTGGTTTCAGATGTGTTGGCACTTCAATGAT
TATTTTTTTCATAAGACTGTTCAGAAGGTTGAATTGATGGGTTGCTTGAGTAGTTGTTGCTTGAGTTAAACTTTG
TATCATTTTAGGCATATTTGGGCATATTTTTTAATCTCAAAAGAAGATTTGGGATAGTTTCAGAGGAGAGTTTTC
TGAACAGACATTTGAAGATGTGAAGAAGGTACCTCTTGCTCGATTTTCCTGAAGACCCCCCATATCCCCCAAGAG
TTAAACTGGAGAAGCTGGAGACTGTGGAGAATATGCAGTTCTATATATGTTTCAGAGTTTTTAAAAAGCATGTGT
ATGTGTGTATCCAGGGATGCAGCATATTGGTATGCTAGGAATACGGTAAAAACTTAAACAAGCTGAACAGTGTTA
TCCTTTTCCTGAGTCGTAAGTCATTCTGCACATCACATGCTGCT

>Zebrafish
GTGCTTGTTCTCAATGGCCGATGAAAATAATCATAAATAATTTGGTGTCATCACCCAGGGAGTTCTTTTAAACAA
GGATAATCAATGTCTTACGCAGGGAATCTAAACACAGATAGCAGATCATTTTGTTCTTGTATGTTATTATAGGAT
CGTCCGTAACTGCATGGACAGAAGCGCTCCGTCTAACTGCCTCCCAGTACGCACTAGCTCAGCAGTAGCAATGAG
CCCACTGCGACCAGGCAGG**GGGGATGAAAAGGA**GCAGGGCGCAAGCGTCAAAAAGAGAAGCAACCCCAAGGAGAA
GTGAAAAAGGCTTCTTTTCGGGGAGGCCTGTTTTGTTTAGAATTGGTCAAGCACAACTACAAAGATGTTGCAGGG
GTACTGCCTTTGAAATTTTCCAAAAGAAAATGATTGTTGGATAAACCGCCCCCAAAACGCCACTCTCTGGCAAAC
GGAGGGAGTGTGACCTATGACCCTCAGTATGAAAGTACAGCGAGACGTACCCCTATATACCACCCTAAGCTCAAC
CCGCCCCGATTTCACCCCCTTTGCTCCACCTCATTCAAGCAAAAAGAGTGAGAGAGAAATTCATGTCATTCATTT
TGCTTCTCATCTCTCTCTCTCTCTCAAGGCTGGGAGGGTGAGTCGTGTCTTAGGAGTCAAGAATTCAATTAGTTA
AAAAAAGGCGAAAAAACAAGAAAGAAAGCAAGAAAAGAAAGAGTCTGCAACAATAAAGCGATAACGGACAGAATG
TGCGTTCCCACACGTTAAATACAGGGCGCTTACTACTGAAAGCACAGCATGGGAGCTATAGCCTCTGACTGCAGG
TGGTGTCCTGAATAATTGCAGTCAATATGCCCCTTCTATGCAGGCTTTGCACAAGCAGATCATTACACACATGCC
ACATACAGGTGTTCCCCAGGCGCATTTGAGATGGAAACTTTTGTTATTCCCTAAAAAGCATTTTTTTTGGGAGCT
GAGAAGTTTGCTTTGAGAGTTTAACTCTGTGGCTGTGGTGTTATGCA

Supplementary Figure 8. Spotted gar and zebrafish CE64. Spotted gar and zebrafish CE64 including the entire sequence corresponding to sequence tested in reference 31. The parts of the sequences that is conserved with mouse and human CE64 is marked in yellow and FIMO hits for phylogenetic footprint #3 in spotted gar and #2 in zebrafish is marked with red, bold and underlined.

Supplementary Table 1. Genomic coordinates of the CRISPR deletions for all founders used in this study.

Strain name	Target CE	Genomic coordinates of deletions MM10	Size of deletion (bp)
58-432	CE58	45,581,399 - 45,582,371	973
59-708	CE59	45,585,002 - 45,585,325	324
61-711	CE61	45,592,927 - 45,594,008	1082
61-713	CE61	45,592,924 - 45,594,025	1102
64-572	CE64	45,615,282 - 45,617,095	1811
64-579	CE64	45,615,283 - 45,617,093	1814
66-541	CE66	45,622,255 - 45,622,840	586
79-529	CE79	45,717,702 - 45,718,193	492
79-681	CE79	45,717,759 - 45,718,349	591
79-281	CE79	45,717,706 - 45,718,198	493
80-622	CE80	45,734,600 - 45,735,569	970
80-615	CE80	45,734,594 - 45,735,569	976

Supplementary Table 2. Genomic coordinates, type of modification and phenotypic outcome for all embryonic CRISPR deletions reported in this study.

Name	Coordinates MM10	Size	Intact MHB	Type of modification
DEL-AC1	45,615,244 - 45,617,008	1765	✗	Deletion
DEL-A1	45,615,265 - 45,616,287	1023	✓	Deletion
DEL-AB1	45,615,282 - 45,616,929	1648	✗	Deletion
DEL-AB3	45,615,282 - 45,616,932	1651	✗	Deletion
DEL64	45,615,283 - 45,617,093	1814	✗	Deletion
DEL-AB2	45,615,790 - 45,616,928	1139	✗	Deletion
DEL-B1	45,616,275 - 45,616,928	654	✗	Deletion
DEL-B8	45,616,221 - 45,616,929<br>45,617,090- 45,617,107	709<br>18	✗	Deletion
DEL-B2	45,616,273 - 45,616,516	244	✓	Deletion
DEL-B4	45,616,517 - 45,616,722	206	✓	Deletion
DEL-B3	45,616,517 - 45,616,928	412	✓	Deletion
DEL-BC1	45,616,542 - 45,617,093	552	✗	Deletion
DEL-BC2	45,616,672 - 45,617,098<br>45,616,100 - 45,617,257	427<br>159	✗	Deletion<br>Inversion
DEL-B7	45,616,720 - 45,616,930	211	✓	Deletion
DEL-BC4	45,616,721 - 45,618,022	1302	✗	Deletion
DEL-B6	45,616,722 - 45,616,936	215	✓	Deletion
DEL-B5	45,616,726 - 45,616,929	204	✓	Deletion
DEL-BC5	45,616,768 - 45,617,102	335	✗	Deletion
DEL-C1	45,616,922 - 45,617,092	171	✗	Deletion
DEL-C6	45,616,923 - 45,616,929	7	✓	Deletion
DEL-C7	45,616,926 - 45,617,534	609	✗	Deletion
DEL-C8	45,616,926 - 45,617,534	609	✗	Deletion
DEL-C9	45,616,927 - 45,617,023	97	✗	Deletion
DEL-C11	45,616,930 - 45,616,938<br>45,616,956 - 45,617,056	304 insertion + 9 deletion<br>101	✗	Indel<br>Deletion
DEL-C2	45,616,930 - 45,616,995	66	✓	Deletion
DEL-C10	45,616,930 - 45,617,055	126	✗	Deletion
DEL-C12	45,616,930 - 45,617,091	162	✗	Deletion
DEL-C13	45,616,930 - 45,617,094	165	✗	Deletion
DEL-C15	45,616,935 - 45,617,257	323	✗	Deletion
DEL-C16	45,616,967 - 45,616,971	5	✓	Deletion
DEL-C17	45,616,968 - 45,617,005	38	✗	Deletion
DEL-C5	45,616,971 - 45,617,007	37	✗	Deletion
DEL-C3	45,616,991 - 45,617,056	66	✓	Deletion
DEL-C18	45,616,999 - 45,617,055	57	✓	Deletion
DEL-C4	45,617,045 - 45,617,146	102	✓	Deletion
DEL-C20	45,617,051 - 45,617,056<br>45,617,088 - 45,617,112	6<br>25	✓	Deletion<br>Deletion
DEL-C19	45,617,051 - 45,617,056<br>45,617,090 - 45,617,108	6<br>19	✓	Deletion<br>Deletion
DEL-C21	45,617,056 - 45,617,081	26	✓	Deletion
DEL-C22	45,617,056 - 45,617,094	39	✓	Deletion

Supplementary Table 3. Primers used for genotyping.

Primer name	Sequence
CE58R-CRISP1F-Surveyor	ACAACCAGGCTGTCTTCCAG
CE58R-CRISP1R-Surveyor	CTCCACCCTACCCCAAGTCT
CE58R-CRISP2F-Surveyor	TGTAGTGCTGTGGTGCTGAG
CE58R-CRISP2R-Surveyor	AGACTCAGGAGGCTAGGGTG
CE59-CRISP1F-Surveyor	AGAGCACACGTGTTCAGACA
CE59-CRISP1R-Surveyor	GGACTGCCCCTCTTGAAAGT
CE59R-CRISP2F-Surveyor	TCAGTTTGGTGTCAGCAGGC
CE59R-CRISP2R-Surveyor	GAACGTGGCTTCAGCTTGTG
CE61-CRISP1F-Surveyor	CTGTGGGAAGGATCGGTCTG
CE61-CRISP1R-Surveyor	TTTGGAGATGACAGGTGGGC
CE61-CRISP2F-Surveyor	ACCATCTGCCACCGAGAATG
CE61-CRISP2R-Surveyor	GAAGAAGGCAGGCACAAAGC
CE64-CRISP1F-Surveyor	AGAGTGACTGGCATCAGTGC
CE64-CRISP1R-Surveyor	GTAGAGGGAAGCATTGGGGG
CE64-CRISP2F-Surveyor	CCTGGGAAAAATGCAGGCAC
CE64-CRISP2R-Surveyor	GAGACCCAGTCCTGACCTCT
CE64-CRISP3F-Surveyor	TTTGGACACACTGACAGGGG
CE64-CRISP3R-Surveyor	GAGGAGGGCGGAATGAAGAG
CE66-CRISP1F-Surveyor	CCAAGGGAACCAAGTGTGGA
CE66-CRISP1R-Surveyor	AGGGGAAAGGGGAGCCATAA
CE66-CRISP2F-Surveyor	TTATGGCTCCCCTTTCCCCT
CE66-CRISP_R2-Surveyor	AGAGGACACAAACAGACGGG
CE79-CRISP1F-Surveyor	CACCAGTCCATGCAGACCAT
CE79-CRISP1R-Surveyor	TCACACACCATACCCCCTGA
CE79-CRISP2F-Surveyor	AATCTCCCTGAGAGTGGCCT
CE79-CRISP2R-Surveyor	ACCTCATTTCCCTGAGGGGT
CE80-CRISP1F-Surveyor	TAAGCATATTGGGCCGGCAA
CE80-CRISP1R-Surveyor	AGGTGAGCAGAAGAGAGGGT
CE80-CRISP2F-Surveyor	CCCAGCTCCTCGCACAATAA
CE80-CRISP2R-Surveyor	TTTTGGCACCTCTCTTGGCA
CE80-CRISP_F2-Surveyor	CCAAGACCTGTGTTGGGTCT
CE80-CRISP_R2-Surveyor	GAAGAGCAATTGCCCAGTGT
CE64-F1	GACCTACACAGGCCGCATTA
CE64-R1	AAGAGACAATGGACCAGCCG
CE64-F2	TCTAGCAATTTGGAGGCGGG
CE64-R2	AAGATTTGGCATGGGAGCCA
64-F3	CCTCGGCACGCCATCTATAC
CE64-R3	AGATACATCGCAAGGCAGCA
Tcf/Lef1 DEL	TGCTAAGTGAGATGAAAGGGGCC

Supplementary Table 4. CRISPR gRNAs used for zygote injections.

gRNA Name	CE Target	Sequence
CE58-CRISP1	58	CCAAGCACCTTGGCCGTGGGAGG
CE58-CRISP2	58	GGGCGGCGTGCGCAGAGCCTTGG
CE59-CRISP1	59	TAACAAGGCGACATAATTACAGG
CE59-CRISP2	59	CTCTCCCCCGCCCCGTACAGGGG
CE61-CRISP1	61	GTACCCACCACGTCAGCGGGAGG
CE61-CRISP2	61	CGGCAACAAGCCCGATCCATGGG
CE64-CRISP1	64	TCAGAAGCTTTACCGCCTAGAGG
CE64-CRISP2	64	TGCCACCTGGTACTTCCCGCAGG
CE64-CRISP3	64	CCTTTCAAATCCGAATGGGGAGG
CE64-CRISP4	64	GTATGATTTCACGTCAGAACAGG
CE64-CRISP5	64	GAGGGAGAAGCAACCCCGATGGG
CE64-CRISP6	64	GGACAACAAGGAAAATCGGCTGG
CE64-CRISP7	64	CGGTAAAGCTTCTGAGAATCTGG
CE64-CRISP8	64	CTCAGCTCTAACTGGGCCTCAGG
CE64-CRISP9	64	GTGCATCTTTGATTAGGTGCAGG
CE64-CRISP10	64	GTGATTGGCAGGCGGGCTATGGG
CE64-CRISP11	64	GTGCATCTTTGATTAGGTGCAGG
CE64-CRISP12	64	TGTGCTAAGTGAGATGAAAGGGG
CE64-CRISP13	64	TTAGCACAGAGAAGAGACAATGG
CE64-CRISP14	64	ACTAGAGGTGATTGGCAGGCGGG
CE64-CRISP15	64	AATAACTAGAGGTGATTGGCAGG
CE64-CRISP16	64	CCAATCACCTCTAGTTATTAAGG
CE66-CRISP3	66	AACAACTCCCTTTGAACCTCCGG
CE66-CRISP6	66	TACAGGGGCAGTGTAGTAATCGG
CE79-CRISP1	79	TGCAGAGCAGCCCGGTAGCTGGG
CE79-CRISP2	79	TATCACGGCCCGGAGCTCAGAGG
CE80-CRISP2	80	CTTCAGGTGCGGGGACACGGGGG
CE80-CRISP6	80	AAGGCAGACCACTCTATCCCTGG

Supplementary Table 5. Cloning primers used for transgenic constructs.

Primer name	Sequence	Target construct
64-1/3 F	TGaagcttCTAGAGGCAGAGCAGCTCAG	Tg(CE64), Tg(DEL-C), Tg(DEL-B)
64-1/3 R	TGctcgagCCCCATTCGGATTTGAAAGGGCCGG	Tg(CE64), Tg(DEL-B)
64-6/3DEL R	TGctcgagCCCCAGATTTTCCTTGTTGTCC	Tg(DEL-C)
64-2/5 F	TGaagcttTCCCTCAGCGATTGCACAG	Tg(64-B)
64-2/5 R	TGctcgagGATTTTCCTTGTTGTCCCTTGTTTG	Tg(64-B)
64-LepOcu F	TGaagcttATGGATGTGGTTGGGAGGTG	Spotted gar 64
64-LepOcu R	TGctcgagGTCTCGCTGTACGCTTATACAAAG	Spotted gar 64

Supplementary Table 6. RT-qPCR primers used for gene expression analysis and their corresponding targets.

Primer name	Sequence	Target gene
#2579_Mm_RT_Dusp6_MKP3_Fwd	GAATGAGAACACTGGTGGAGAGTCG	Dusp6
#2580_Mm_RT_Dusp6_MKP3_Rev	ACTCGGCCTGGAACTTACTGAAGC	
#2569_Mm_RT_En1_Fwd	TGGGTCTACTGCACACGCTATTCG	En1
#2570_Mm_RT_En1_Rev	GCCGCTTGTCTTCCTTCTCGTTC	
#2555_Mm_RT_En2_Fwd	GCTATTCTGACCGGCCTTCTTCAG	En2
#2556_Mm_RT_En2_Rev	GGTACCTGTTGGTCTGAAACTCAGC	
#2567_Mm_RT_Etv4_PEA3_Fwd	AGGAAGCCACCACTCCCCTACCAC	Etv4
#2568_Mm_RT_Etv4_PEA3_Rev	GGGACTTGATGGCGATTTGTCTG	
RTqPCR_Etv5_Fwd	TGAGCAGTTTGTCCCAGATTTTCAG	Etv5
RTqPCR_Etv5_Rev	CTGTGCAGCTCCCGTTTGATCTTG	
#2529_Mm_RT_Fbxw4 _Fwd	CCCACAACAAGCTGTTCCAGTCAC	Fbxw4
#2530_Mm_RT_Fbxw4 _Rev	TACACACCATCCAGACCGAAGACC	
#2599_Mm_RT_FGF17_Fwd	AAGAGGGGCAAGCTGATTGGGAAG	Fgf17
#2600_Mm_RT_FGF17_Rev	AAAGCCATGAACCAGCCCTCGTG	
#2601_Mm_RT_FGF18_Fwd	GGCGAGGACGGGGACAAGTATG	Fgf18
#2602_Mm_RT_FGF18_Rev	CCGGACTTGACTCCCGAAGGTATC	
#2531_Mm_RT_Fgf8_Fwd	TATCGGTCTCCACAATGAGCTTCG	Fgf8
#2532_Mm_RT_Fgf8_Rev	CCTGGCCAACAAGCGCATCAAC	
Fwd_SYBR_FgFR1	TCTGGCCTCTACGCTTGC	Fgfr1
Rev_SYBR_FgFR1	AGGATGGGAGTGCATCTGA	
#2561_Mm_RT_Gbx2_Fwd	GCTCGCTGCTCGCTTTCTCTGC	Gbx2
#2562_Mm_RT_Gbx2_Rev	GCTGTAATCCACATCGCTCTCCAG	
#2568_Mm_RT_Etv4_PEA3_Rev	GGGACTTGATGGCGATTTGTCTG	Bmp4
#2569_Mm_RT_En1_Fwd	TGGGTCTACTGCACACGCTATTCG	
Hprt_mRNA_F	CTTCCTCCTCAGACCGCTTTT	Hprt
Hprt_mRNA_R	CATCATCGCTAATCACGACGC	
#2571_Mm_RT_Lmx1b_Fwd	AGATGAAGAAGCTGGCCCGGAGAC	Lmx1b
#2572_Mm_RT_Lmx1b_Rev	CTCCATGCGGCTTGACAGAACCTC	
#2557_Mm_RT_Otx2_Fwd	CTGCATCCCTCCGTGGGCTACC	Otx2
#2558_Mm_RT_Otx2_Rev	CGTCGAGCTGTGCCCTAGTAAATG	
#2507_Mm_RT_Pax2_Fwd	CGAGGGCATCTGCGATAATGAC	Pax2
#2508_Mm_RT_Pax2_Rev	CTGCTGAACTTTGGTCCGGATG	
Fwd_SYBR_Pax5	ACGCTGACAGGGATGGTG	Pax5
Rev_SYBR_Pax5	GGGGAACCTCCAAGAATCAT	
mPax6-qpcr-fwd	CAAACACACATGAACAGTCAGC	Pax6
mPax6-qpcr-rev	ACTTGGACGGGAACTGACAC	
Fwd_SYBR_MmPGK1	TACCTGCTGGCTGGATGG	PGK1
Rev_SYBR_MmPGK1	CACAGCCTCGGCATATTTCT	
#2565_Mm_RT_Sp8_Fwd	TCGACGATTTCGAAGGGGAACG	Sp8
#2566_Mm_RT_Sp8_Rev	TGGGGCTGCCGATCTTATTACAGG	
#2551_Mm_RT_Spry1_Fwd	GGTCATAGGTCAGATCGGGTCATCC	Spry1

#2552_Mm_RT_Spry1_Rev	TGGGTGGGGTCCTCTTTCAAGG	
#2549_Mm_RT_Spry2_Fwd	AAGGGAGAGGGGTTGGTGCAAAG	Spry2
#2550_Mm_RT_Spry2_Rev	CCATCAGGTCTTGGCAGTGTGTTC	
#2577_Mm_RT_Shh_Fwd	CCCCAATTACAACCCCGACATC	Shh
#2578_Mm_RT_Shh_Rev	GGCATTTAACTTGTCTTTGCACCTCTG	
#2559_Mm_RT_Wnt1_Fwd	CCAACAGTAGTGGCCGATGGTG	Wnt1
#2560_Mm_RT_Wnt1_Rev	CAGACTCTTGGAATCCGTCAACAGG	
