# Supplementary material for: Dissection of the Fgf8 regulatory landscape by in vivo CRISPR-editing reveals extensive intra- and inter-enhancer redundancy
Source: Nat Commun. 2021 Jan 19;12:439. doi: 10.1038/s41467-020-20714-y (PMC7815712; doi:10.1038/s41467-020-20714-y)
Supplement: Supplementary file 3 — Description of Additional Supplementary Files [file 41467_2020_20714_MOESM3_ESM.pdf]

Description of Additional Supplementary Files

Supplementary Movie 1. DEL64 mutants display severe hypoplasia of the midbrain and cerebellum. Tomographic sections from e18.5 mutant brain generated by optical projection tomography. Note the absence of all the MHB-derived structures: the superior colliculus, inferior colliculus, isthmus, and cerebellum.

Supplementary Movie 2. DEL79 mutants display normal brain anatomy. Tomographic sections from e18.5 mutant brain generated by optical projection tomography. Note the normal appearance of all MHB derived structures: the superior colliculus, inferior colliculus, isthmus, and cerebellum.

Supplementary Movie 3 DEL80 mutants display normal brain anatomy. Tomographic sections from e18.5 mutant brain generated by optical projection tomography. Note the normal appearance of all MHB derived structures: the superior colliculus, inferior colliculus, isthmus, and cerebellum.

Supplementary Movie 4. DEL79-80 mutants display normal brain anatomy. Tomographic sections from e18.5 mutant brain generated by optical projection tomography. Note the normal appearance of all MHB derived structures: the superior colliculus, inferior colliculus, isthmus, and cerebellum.
