# Supplementary material for: Dissection of the Fgf8 regulatory landscape by in vivo CRISPR-editing reveals extensive intra- and inter-enhancer redundancy
Source: Nat Commun. 2021 Jan 19;12:439. doi: 10.1038/s41467-020-20714-y (PMC7815712; doi:10.1038/s41467-020-20714-y)
Supplement: Supplementary file 8 — Reporting summary [file 41467_2020_20714_MOESM8_ESM.pdf]

nature research

Corresponding author(s): Francois Spitz

Last updated by author(s): Nov 13, 2020

Reporting Summary

Nature Research wishes to improve the reproducibility of the work that we publish. This form provides structure for consistency and transparency in reporting. For further information on Nature Research policies, see our Editorial Policies and the Editorial Policy Checklist.

Statistics

For all statistical analyses, confirm that the following items are present in the figure legend, table legend, main text, or Methods section.

n/a	Confirmed	
☐	☒	The exact sample size (n) for each experimental group/condition, given as a discrete number and unit of measurement
☐	☒	A statement on whether measurements were taken from distinct samples or whether the same sample was measured repeatedly
☐	☒	The statistical test(s) used AND whether they are one- or two-sided<br>*Only common tests should be described solely by name; describe more complex techniques in the Methods section.*
☒	☐	A description of all covariates tested
☒	☐	A description of any assumptions or corrections, such as tests of normality and adjustment for multiple comparisons
☐	☒	A full description of the statistical parameters including central tendency (e.g. means) or other basic estimates (e.g. regression coefficient) AND variation (e.g. standard deviation) or associated estimates of uncertainty (e.g. confidence intervals)
☐	☒	For null hypothesis testing, the test statistic (e.g. F, t, r) with confidence intervals, effect sizes, degrees of freedom and P value noted<br>*Give P values as exact values whenever suitable.*
☒	☐	For Bayesian analysis, information on the choice of priors and Markov chain Monte Carlo settings
☒	☐	For hierarchical and complex designs, identification of the appropriate level for tests and full reporting of outcomes
☒	☐	Estimates of effect sizes (e.g. Cohen's d, Pearson's r), indicating how they were calculated

Our web collection on statistics for biologists contains articles on many of the points above.

Software and code

Policy information about availability of computer code

Data collection	Drishti v2.6.3 for 3D renderings/movies
Data analysis	NRecon version 1.6.9.18 (Skyscan) software for reconstruction of tomographic sections<br>Prism 8 (GraphPad)

For manuscripts utilizing custom algorithms or software that are central to the research but not yet described in published literature, software must be made available to editors and reviewers. We strongly encourage code deposition in a community repository (e.g. GitHub). See the Nature Research guidelines for submitting code & software for further information.

Data

Policy information about availability of data

All manuscripts must include a data availability statement. This statement should provide the following information, where applicable:

- Accession codes, unique identifiers, or web links for publicly available datasets
- A list of figures that have associated raw data
- A description of any restrictions on data availability

The data generated and analyzed during this study are include in the published article (and in its supplementary files). Additional details regarding the constructions used and the transgenic animals are available from the corresponding author.

Field-specific reporting

Please select the one below that is the best fit for your research. If you are not sure, read the appropriate sections before making your selection.

- [x] Life sciences
- [ ] Behavioural & social sciences
- [ ] Ecological, evolutionary & environmental sciences

For a reference copy of the document with all sections, see nature.com/documents/nr-reporting-summary-flat.pdf

Life sciences study design

All studies must disclose on these points even when the disclosure is negative.

Sample size	Sample size was not predetermined, but follow recommended guidelines and practice of the field for comparison of transgenic constructs (minimal= 3 - Haruyama et al. 2009 - 10.1002/0471143030.cb1910s42). This is limiting of course the statistical power of the comparisons, as mentioned in the manuscript
Data exclusions	no data was excluded
Replication	Experimental findings were replicated as follows. Pattern of transgenic activities were determined by analyzing independent transgenic embryos (same construct, but different genomic insertions, different day of assays - at least two days). As expected, some variation is observed between transgenic animals for the same construct, due to position effects, which can lead in a insertion-site-specific manner to addtional expression domains and/or total silencing of the transgene. We therefore only consider as the domain of expression of the transgene, the specific regions in which we detected the transgenes in multiple independent replicates. For each transgenic construction, we indicate both the total number of replicates and the number of replicates in which the expression domain (e.g. limb AER, MHB) is detected (y). In addition, we provide photos (Supplementary Figure 6) of all the replicate embryos showing expression.<br>RT-qPCR assays were performed at least twice and on multiple independent samples (from distinct embryos and litters).
Randomization	not applicable
Blinding	For the LacZ staining and phenotypic analysis, the genotypes of the animals (transgenic or not) were determined AFTER their analysis.<br>For RT-qPCR experiments, the investigators were not blind to the samples, as the samples were collected and analyzed by the same person.

Reporting for specific materials, systems and methods

We require information from authors about some types of materials, experimental systems and methods used in many studies. Here, indicate whether each material, system or method listed is relevant to your study. If you are not sure if a list item applies to your research, read the appropriate section before selecting a response.

Materials & experimental systems

n/a	Involved in the study
x	Antibodies
x	Eukaryotic cell lines
x	Palaeontology and archaeology
	x Animals and other organisms
x	Human research participants
x	Clinical data
x	Dual use research of concern

Methods

n/a	Involved in the study
x	ChIP-seq
x	Flow cytometry
x	MRI-based neuroimaging

Animals and other organisms

Policy information about studies involving animals; ARRIVE guidelines recommended for reporting animal research

Laboratory animals	The study is based on the analysis of transgenic mouse embryos (strain background C57Bl/6 for Crisp/Cas9 and FVB for transgenic pronuclear injection). Embryos were collected at 8 to 18 days in utero (no selection/identification of sex), with morning after successful mating (judge by the presence of a vaginal plug) taken as day 0 (as embryos are developing in utero, they are minimally influenced by temperature, details of day/night cycles (set on a 12/12) etc ...)
Wild animals	the study involved NO wild animals
Field-collected samples	the study did not involved field-collected samples
Ethics oversight	The experimental protocols and plans were reviewed and approved by the Animal Experiment Institutional Committees of the European Molecular Biology Laboratory (Heidelberg- Germany) and Institut Pasteur (Paris- France), where the experiments were conducted.

Note that full information on the approval of the study protocol must also be provided in the manuscript.
